# Access to care issues adversely affect breast cancer patients in Mexico: oncologists’ perspective

**DOI:** 10.1186/1471-2407-14-658

**Published:** 2014-09-09

**Authors:** Yanin Chavarri-Guerra, Jessica St Louis, Pedro ER Liedke, Heather Symecko, Cynthia Villarreal-Garza, Alejandro Mohar, Dianne M Finkelstein, Paul E Goss

**Affiliations:** MGH-Avon International Breast Cancer Program, Massachusetts General Hospital, Boston, MA USA; Hemato-Oncology Department, National Institute of Medical Sciences and Nutrition Salvador Zubirán, Mexico City, Mexico; Biostatistics Center, Massachusetts General Hospital, Boston, MA USA; Medical Oncology and Breast Cancer Departments, National Cancer Institute, Mexico City, Mexico; Biomedical Research Unit in Cancer, National Autonomous University of Mexico, Mexico City, Mexico; National Cancer Institute, Mexico City, Mexico; Harvard Medical School, Boston, MA USA; Massachusetts General Hospital Cancer Center, 55 Fruit Street, Lawrence House, LRH-302, Boston, Massachusetts 02114 USA

**Keywords:** Breast cancer, Socioeconomic disparities, Mexico, Access to care, Patterns of care, Survey

## Abstract

**Background:**

Despite recently implemented access to care programs, Mexican breast cancer (BC) mortality rates remain substantially above those in the US. We conducted a survey among Mexican Oncologists to determine whether practice patterns may be responsible for these differences.

**Methods:**

A web-based survey was sent to 851 oncologists across Mexico using the Vanderbilt University REDCap database. Analyses of outcomes are reported using exact and binomial confidence bounds and tests.

**Results:**

138 participants (18.6% of those surveyed) from the National capital and 26 Mexican states, responded. Respondents reported that 58% of newly diagnosed BC patients present with stage III-IV disease; 63% undergo mastectomy, 52% axillary lymph node dissection (ALND) and 48% sentinel lymph node biopsy (SLNB). Chemotherapy is recommended for tumors > 1 cm (89%), positive nodes (86.5%), triple-negative (TN) (80%) and HER2 positive tumors (58%). Trastuzumab is prescribed in 54.3% and 77.5% for HER2 < 1 cm and > 1 cm tumors, respectively. Tamoxifen is indicated for premenopausal hormone receptor (HR) positive tumors in 86.5% of cases and aromatase inhibitors (AI’s) for postmenopausal in 86%. 24% of physicians reported treatment limitations, due to delayed or incomplete pathology reports and delayed or limited access to medications.

**Conclusions:**

Even though access to care programs have been recently applied nationwide, women commonly present with advanced BC, leading to increased rates of mastectomy and ALND. Mexican physicians are dissatisfied with access to appropriate medical care. Our survey detects specific barriers that may impact BC outcomes in Mexico and warrant further investigation.

**Electronic supplementary material:**

The online version of this article (doi:10.1186/1471-2407-14-658) contains supplementary material, which is available to authorized users.

## Background

Breast cancer (BC) is the leading cancer among women worldwide [1, 2]. In Mexico, BC incidence has been increasing in recent decades with 8,428 cases reported in 2009. This reflects a national incidence of 15 per 100,000 women compared with 76 per 100,000 women in the US, although figures in Mexico are underreported due to a lack of a National Cancer Registry [3]. Since 2006 it has been the leading cause of cancer mortality in Mexican women, accounting for 14% of all female cancer-related deaths [4]. While the incidence of BC in Mexico is lower than the US, the ratio mortality/incidence in Mexico is almost the double that in the US (37% vs. 18.7%) [5].

Recent changes in Mexican health care policies have incorporated programs addressing access to early breast cancer (EBC) diagnosis and treatment [6]. The implementation of the Seguro Popular (SP), the Mexican Health Insurance in 2003, was part of health reform intended to provide health coverage for the poor and uninsured [7]. SP also includes protection of the poor from “catastrophic health expenditures”, such as those commonly resulting from a diagnosis and subsequent treatment of BC [7]. In 2011, the BC protocol for SP included: diagnostic workup for EBC; local and systemic treatment, such as breast and axillary surgery (breast conservation surgery/mastectomy and SLNB/ALND); and, when appropriate, adjuvant radiation therapy, chemotherapy, endocrine therapy (ET) and trastuzumab (for HER2 positive BC) [8, 9]. Although the SP program appears to have had a significant impact on access to BC care, there remains a paucity of data as to whether the program has yet impacted the incidence and mortality of BC [10].

The aim of the survey reported here, was to assess patterns of current care among a spectrum of oncologists currently providing clinical care to newly diagnosed BC patients in Mexico. Assessment of physician’s decisions under scenarios of free access to care versus current access to care was our means of examining how socioeconomic factors impact patient care.

## Methods

A list of oncologists was obtained from the Mexican Oncology Board [11]. There were a total of 983 oncologists listed within the Mexican Oncology Board who had an available email address (including medical oncologists, oncologic surgeons, gynecologic oncologists, radiotherapists, and pediatric oncologists). From the MGH-Avon International Breast Cancer Program in Boston, a web-based survey was sent to 851 oncologists (excluding 132 pediatric oncologists). Non-responders were sent email reminders to complete the survey 2, 3 and 7 weeks after the initial invitation. No incentives were offered to participating physicians.

The survey consisted of 35 questions which were divided into sections that addressed: physician demographics; BC patient demographics and clinical presentation; details of pathology reports and; patterns of treatment for patients with EBC (Additional file 1: Figure S1). Questions addressing systemic therapy could be answered with more than one option. Anonymous responses were entered directly by the physicians into the Research Electronic Data Capture (REDCap), a secure Vanderbilt University database, for analysis [12]. All responses were tabulated and analyzed using Stata Statistical Software: Release 12. Confidence bounds on proportions were derived from Chi-squared or exact distributions depending on sample size. Exact binomial proportion confidence intervals were used to compare distribution of responses. The study was approved by the Partners Human Research Committee and complied with the Declaration of Helsinki.

## Results

### Demographics

**Figure 1:**
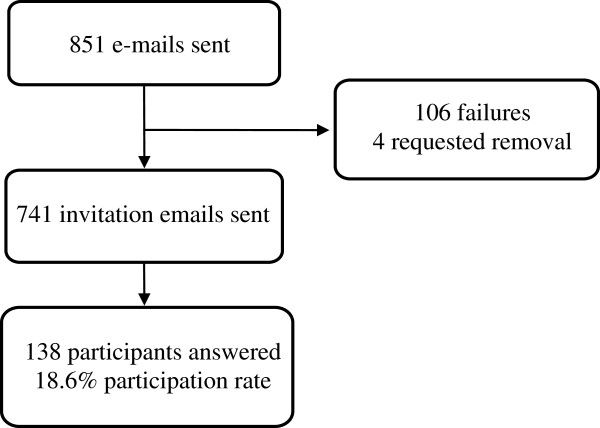
**Flow of participants.** 851 members of the Mexican Oncology Board were invited to participate in the online survey. 106 email addresses experienced delivery failures, and 4 individuals requested removal from further survey invitations. Subsequently, 741 invitation emails were sent, and 138 participants answered the survey.

One hundred and thirty-eight participants answered the web-survey, representing an 18.6% response rate (Figure 1). One hundred and six email addresses experienced delivery failures. Of the 138 responders, 129 (93%) completed the questionnaire. Two responders reported that they did not practice medicine in Mexico and were therefore excluded from our analyses. Table 1 displays the demographics of survey participants.

**Table 1:** **Demographic characteristics of survey participants**

Characteristics of physicians surveyed	Number (Percentage)
***Gender***	
Male	79 (58%)
Female	57 (42%)
***Age***	
<40 years	59 (43%)
40-65 years	74 (54%)
>65 years	3 (2%)
***Years since Medical Graduation***	
1-10	56 (41%)
> 10	80 (59%)
***Specialty***	
Medical Oncology	43 (32%)
Surgical Oncology	79 (58%)
Breast Surgeon	7 (5%)
Other	7 (5%)
***Location of Primary Clinical Practice***	
Academic medical center	36 (26.9%)
Public hospital/clinic	64 (47.8%)
Philanthropic hospital/clinic	2 (1.5%)
Private hospital/clinic	27 (20.1%)
Other	5 (3.7%)
***Geographic Area of Practice***	
Urban Center	130 (95.6%)
Suburban	5 (3.7%)
Rural	1 (0.7%)
***Regional Distribution***	
Northern Mexico	32 (24%)
Central Mexico	83 (62%)
Southern Mexico	18 (14%)
***Form of Patient Payment (Estimates)***	
Out of Pocket (OOP)	20%
Private Insuranc	20%
Public Insurance	51%
Not Insured and Not OOP	8%
Other	1%

### Breast cancer diagnosis

The stage of disease at presentation was 42% for Stage I-II, 44% for stage III and 14% for stage IV. Physicians reported that tumor size, tumor grade, vascular invasion, tumor margin status, lymph node analysis, estrogen receptor (ER 88.4%), progesterone receptor (PR 87.7%), and HER2 (87.7%) receptor results were standard elements of pathology reports (Table 2). The physicians reported that HER2 analysis was performed by either immunohistochemistry (93.5%) or fluorescent in situ hybridization (59.4%). Of the physicians that routinely tested for HER2, 48% reported that testing was done in their local hospital, while 49% reported that testing was performed in a central regional lab. Four percent of physicians reported that HER2 was not routinely analyzed in their practice.

**Table 2:** **Characteristics available on pathology reports**

*Pathologic characteristics*	*Percentage*
	*n = 136*
Tumor size	96.4
Tumor grade	97.8
Presence/absence of vascular invasion	94.2
Margins	93.5
Lymph node analysis	97.1
Estrogen receptor	88.4
Progesterone receptor	87.7
HER2/neu	87.7

### Patterns of local therapy

Physicians reported mastectomy rates of 63% and lumpectomy rates of 37% in localized breast cancer patients. In women without palpable lymph nodes, physicians reported SLNB and ALND rates of 48% and 52% respectively. Ninety-four percent of physicians reported that adjuvant radiotherapy is available. Of those, 92.1% reported that patients routinely receive daily-fractionated radiotherapy for duration of 5–6 weeks regardless of the type of surgery or clinical stage.

### Patterns of systemic therapy

Physicians reported that neoadjuvant therapy is recommended in 88.4% of their patients that present with stage III, 27.8% in patients with stage II, and 4.7% of patients with stage I. An average time interval of 3–12 weeks between definitive surgery and adjuvant chemotherapy was reported by 86.6% of the physicians. Others reported time intervals of less than 3 weeks (11.9%) and greater than 12 weeks (1.5%).

### Management of hormone receptor positive breast cancer

**Figure 2:**
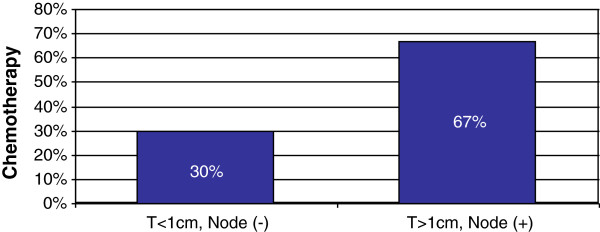
**Patterns of chemotherapy for ER + disease.** 30% of physicians recommended chemotherapy for patients with ER + tumors of less than 1 cm in size and negative nodes, while 67% of physicians recommended chemotherapy for patients with tumors greater than 1 cm in size and positive nodes.

When treating patients with low risk HR + BC (defined by HER2 negative, less than 1 cm tumors and negative lymph nodes), 65% of physicians recommend only ET, 26.9% recommend ET and chemotherapy,7.1% only chemotherapy and <1% ET and Oncotype assessment (Figure 2). The most commonly prescribed regimen is anthracycline chemotherapy in 73.8%, followed by taxane in 35.7%, and anthracycline-taxane regimens in 32.2%.

When treating patients with high risk HR + BC (defined by HER2 negative, greater than 1 cm tumors, positive lymph nodes), 52% of physicians recommend combination ET and chemotherapy and 48% recommend chemotherapy only (Figure 2). The most commonly prescribed chemotherapy is anthracycline-taxane (85.2%), followed by anthracycline (19.3%), taxane (14.7%), taxane-platinum (<1%), gemcitabine (<1%) and bevacizumab (<1%).

The patterns of ET prescribed for premenopausal women with HR + tumors are: tamoxifen (55.5%), tamoxifen and ovarian suppression (OS) (19%), AI’s only (14.2%), combination of tamoxifen, OS, and AI’s (11.9%), AI’s and OS (4.7%), and OS only (3.9%). For postmenopausal women, the most common ET prescribed was AI’s only in 42.1%, followed by tamoxifen and AI’s in 38.2%, tamoxifen only in 14%, OS, tamoxifen, and AI’s (5.4%) and fulvestrant (<1%). The average duration of therapy prescribed in premenopausal and postmenopausal women for tamoxifen was 3.9 years in both groups, and for AI’s 1.5 and 4.3 years, respectively.

### Management of triple negative breast cancer

**Figure 3:**
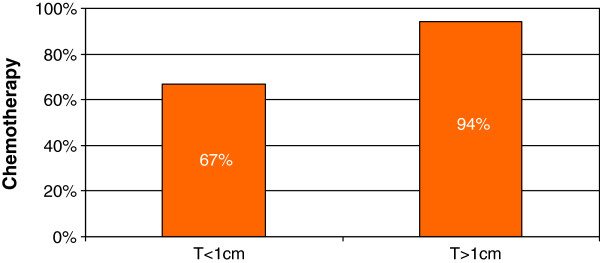
**Patterns of chemotherapy for TN disease.** 67% of physicians recommended chemotherapy for patients with TN tumors of less than 1 cm in size, while 94% of physicians recommended chemotherapy for patients with TN tumors greater than 1 cm in size.

With respect to TNBC, with tumors less than 1 cm and negative lymph nodes, 30.2% recommend no adjuvant therapy, 37.3% recommend anthracycline-taxane chemotherapy, 24.6% anthracycline therapy, and 5.6% taxane chemotherapy (Figure 3).

For patients with TNBC with tumors greater than 1 cm and negative lymph nodes, 3.2% of physicians recommend no adjuvant therapy, 64% recommend anthracycline-taxane chemotherapy, 22.4% anthracycline therapy, and 8% taxane treatment.

### Management of HER2-positive (HER2 +) breast cancer

**Figure 4:**
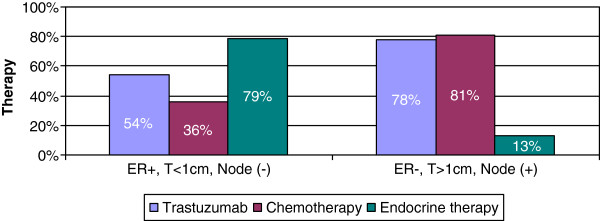
**Patterns of therapy for HER2+ disease.** For patients with tumors that are HER2+, ER+, less than 1 cm in size and node negative, 54% of physicians recommended trastuzumab, 36% recommended chemotherapy and 79% recommended endocrine therapy. For patients’ tumors that are HER2+, ER-, greater than 1 cm in size, and node negative, 78% of physicians recommended trastuzumab, 81% recommended chemotherapy, and 13% recommended endocrine therapy. Participants could select multiple therapy options to describe their treatment of HER2+ patients.

Physicians treating patients with HER2+ and HR + tumors that are less than 1 cm with negative lymph nodes recommend ET (79%), trastuzumab (54.3%), and chemotherapy (36.2%) (Figure 4).

For patients with HER2+ and HR negative tumors that are greater than 1 cm with positive lymph nodes, 13% of physicians recommend ET, 77.5% trastuzumab, and 81.2% chemotherapy (Figure 4).

Forty-eight percent of the physicians surveyed reported that in the last year there were instances where they recommended adjuvant trastuzumab to a patient that ultimately did not receive it. They estimated that non-receipt of trastuzumab occurs in 14.4% of their cases. The most commonly reported reasons for not receiving trastuzumab were: lack of financial coverage for trastuzumab under public health care (26.8%), high out-of-pocket cost (31.2%), and patient co-morbidities or toxicity concerns (10.9%). Other reasons, such as patient refusal (6.5%), lack of coverage for trastuzumab under private health care (8%), referral to a clinical trial (0.7%), alternative opinion of another practitioner (4.3%), and inability to make the trips and visits necessary for treatment (2.9%) were also cited as reasons for not receiving trastuzumab.

### Quality of breast cancer care

Twenty-five percent of physicians changed their treatment recommendations in at least one of the clinical scenarios if offered free access to any medication (95% CI, 0.16 to 0.37). Throughout this series of questions, the scenario of free access to treatment led 68% of physicians to change their decision once, 20% twice, and 12% three times.

With free access to therapy, physicians changed their recommendations most frequently on questions regarding ET for HR + disease (95% CI, 0.04 to 0.16), chemotherapy for TN disease (95% CI, 0.03 to 0.14), and trastuzumab for HER2+ disease (95% CI, 0.05 to 0.15).

A substantial number of physicians reported that they were unable to provide the best treatments for their patients (23.8%). This was attributed to delays in pathology reports (43.3%), omission of important prognostic and predictive information on pathology reports (46.7%), restrictions for prescribing standard chemotherapy (56.7%), delay in receipt of chemotherapy after prescription (43.3%), limitations in prescribing standard ET (40%), restrictions in prescribing trastuzumab for HER2+ patients (63.3%), and high workload (40%).

### Access to clinical trials

With respect to clinical trials, 47.2% of physicians report that there are BC clinical trials that are actively enrolling patients at or near their primary practice. Of the physicians who report having active clinical trials at or near their primary practice, 68.3% indicated that they regularly recommend their patients for trial enrollment.

## Discussion

The goal of our survey was to ascertain patterns of practice in Mexico from clinical oncologists in an attempt to obtain treating physicians’ diagnostic and treatment tools for managing BC. These answers can help derive potential causes of high Mexican BC mortality rates and suggest ways of improving the system deficiencies. Our survey was also conducted in a period post-implementation of the SP, a health care reform in Mexico aspiring to bring universal health care to the population.

We acknowledge that this study had several limitations. First, the response rate was low (18.6%), which might not be the most accurate representation of Mexican oncologists. However, we found this group to be geographically distributed in a statistically comparable way to the overall geographic distribution of physicians practicing within the Mexican Society of Oncology (p = 0.652) [11]. The physicians we surveyed likely represented a typical cross section of treating oncologists because they come from a spectrum of private and public health care systems as well as urban and diverse provincial centers. Importantly, in the subgroup who did respond, we found substantive concern about the impact of socioeconomic barriers on access to care and physician decision-making. The second limitation of our survey was its web-based platform, making it inaccessible to physicians without Internet access, which may have resulted in failure to capture the problems that remote community physicians face. Third, we are aware of a non-response bias, which has been apparent by the fact that the majority of surveyed oncologist reported to have radiotherapy services access. However it is well recognized that radiotherapy is not accessible in several regions of the country [9]. Therefore, we believe the major reason for large number of non-responders to our survey was limited access to the Internet, which mainly represent the oncologists from less specialized centers and underdeveloped areas in the country.

If anything our survey results may understate the discrepancies seen in Mexico in comparison to western countries. It is thus unwise to make definitive conclusions about patterns of breast cancer care in all of Mexico from our survey results, although the true situation is likely to be worse than our results suggest due to under representation of physicians in underdeveloped and disadvantaged areas of the country. We plan a revised survey in the future using this report as our first benchmark.

Despite the implementation of SP and other programs intended to increase early detection, surveyed physicians continue to see newly diagnosed patients presenting with late stage BC [13, 14], and our survey results appear to affirm that as in other low- and middle-income countries, mortality rates in Mexico are largely driven by late, advanced stage of disease at presentation. Specifically surveyed physicians reported that the majority of their newly diagnosed patients present with stage III or IV disease (58%) which is in sharp contrast to the United States, where only 5-12% of white women and 16-20% of Hispanic women living in the US, present with late stage disease at clinical presentation of BC [15, 16].

Closer scrutiny of the clinical pathways addressed in this survey is merited. In terms of early detection, low participation in screening programs persists despite implementation of early detection programs. This is probably aggravated by socio-cultural factors that foster delayed times to diagnosis and limited access to existing specialized centers, which has been exemplified by a survey conducted at the US-Mexico border where Mexican women living in Mexico were less likely to have a screening mammogram than Latinas living in the US [5, 17, 18]. Future policies must incorporate methods to improve early detection focusing on access to care and addressing psychosocial factors that lead women to seek care at late stages of the disease, such as cultural barriers, lack of BC awareness in the general population as well as by primary health care providers, and in many areas where there are persistent deficiencies in mammographic screening programs [19].

Our survey found that rates of total mastectomy (63%) and ALND (52%) are approximately double those seen in the US (33% and 36%, respectively). This in part probably reflects more advanced stage at diagnosis, which is more commonly seen in Mexico and often precludes conservative surgery [9, 20, 21]. In terms of local disease control through radiation therapy, although 94% of physicians reported availability of adjuvant radiotherapy for their patients, the high rates of mastectomy and ALND in patients without palpable nodes may partially be due to the known lack of available and centralized radiation oncology specialists in Mexico, as well as the costs associated with SLNB procedures [9].

In terms of adequate, available pathology reporting, the crucial component of clinical decision-making, the majority (88%) of surveyed physicians report receiving pathology reports that meet international recommendations. However, this rate of adequacy is still lower than that reported in US (98.5%) [16], and contradictory with the statement above, nearly a quarter of physicians claim that they are unable to provide optimal clinical care to their patients, mainly due to delays in pathology reports, and omission of important prognostic and predictive information. These delays and omissions may have a significant impact on the physician’s ability to facilitate appropriate treatments in a timely manner. We and others, have shown that pathology reporting errors are a commonly encountered problem worldwide, exemplified by the 20% discrepancy in results of HER2 biomarker over-expression assessment between central and local laboratories, which can lead to very costly under- or overtreatment of women with HER2 + BC [22]. Quality control programs within BC pathology laboratories urgently need to be addressed in Mexico, as well as internationally.

In terms of adjuvant ET, a key measure in improving mortality risk is to provide appropriate treatment for young premenopausal women, who are disproportionately represented in a young population such as Mexico [14]. The most common ET for women with HR + tumors is tamoxifen without OS for premenopausal women and AI’s for postmenopausal women, comparable to standard practice in the US. Worryingly however, some 14% of physicians in our survey recommended AI’s as mono-therapy for premenopausal women, which is known to be ineffective, may cause unwanted pregnancies, and even compromise outcome results [23]. Furthermore, five percent of physicians also recommended costly OS for postmenopausal women, despite its complete ineffectiveness. Urgent education programs are needed to avoid the increased costs and morbidities associated with these improper practices [24]. Adjuvant chemotherapies are usually recommended by the physicians we surveyed for high-risk patients with larger tumors, node positive tumors, and TN and HER2+ tumors. These are comparable to adjuvant chemotherapy practice patterns in the US reported in 2002, where nearly 80% of physicians prescribed chemotherapy for node positive disease [25]. Again, it is troubling that one quarter of the physicians we surveyed reported concerns about being able to provide the best treatment for their patients. More than half attributed this failure to restricted availability of the optimal chemotherapy choice.

The accelerating pace of scientific development in BC has been increasing during the last decades, which needs evaluation through clinical trials. Our results indicate that Mexican oncologists are well sensitized to refer patients to clinical trials enrollment and comparable to US rate (Mexican surveyed oncologist 68% vs. US oncologist 56.7-71%) [26, 27].

Results of our survey highlight the important problem of ongoing financial barriers to optimal clinical care of BC patients in Mexico. This problem is exemplified by the result that 25% of the physicians we surveyed changed their treatment recommendations when presented with a hypothetical treatment scenario involving free access to medications. While cost constraints are a universal consideration in cancer care, physicians in Mexico are forced to weigh difficult financial decisions when considering how best to treat their patients. These restraints frequently end up hindering their ability to deliver standard, guideline-based care. A specific example of this is treatment decisions in HER2+ BC. Although a majority of physicians report testing patients for tumor HER2 positivity, 48.4% stated that there are instances when they have recommended adjuvant trastuzumab to a patient and that the patient has not been able to receive it. The most common reasons cited for this is lack of coverage under public health care and high out-of-pocket cost. As failure to treat this specific sub-type of BC will contribute to disproportionately higher mortality rates, this finding in our survey is particularly troubling and highlights that despite the implementation of SP, cancer care is still not optimal.

Our survey highlights significant patterns of practice among current oncologists which are likely to have an adverse impact on patients’ outcomes. The patterns of care have been show to be amenable to change. Implementation and monitoring of practice guidelines within Mexico, implementation of tumor board educational telemedicine and other interventions are some examples of measures that may be helpful.

## Conclusions

The SP health system reform in Mexico is widely recognized as both an outstanding and challenging strategy designed to ameliorate a previously inefficient health care system. However, health systems often need to be modified and tailored over time in response to detection of nation’s specific health care needs [28].

Currently, with the implementation of the SP and the social security health care services, “virtually” every woman in Mexico should have warrantee access to BC diagnosis and treatment. However, distribution of specialized centers and physicians, as well as socio-cultural factors, might contribute to the persistence of poor access to timely, adequate and complete BC diagnosis and treatment for certain populations identified by this survey. This potential barrier to care should be investigated further to grasp the extent of this issue and also must be acknowledged by health authorities. Our survey has highlighted an urgent need for improving education among physicians in order to promote judicious use of existing resources. The intention of this survey is to highlight opportunistic areas for improvement within the BC care chain, which are relevant not only to the oncology authorities, but also could serve as a model for addressing access issues in the treatment of other non-communicable diseases in Mexico and other developing countries.

## Electronic supplementary material

Additional file 1: Figure S1: Oncology physician patterns of practice survey. (DOCX 23 KB)

## Abbreviations

BC: Breast cancer
EBC: Early breast cancer
ALND: Axillary node dissection
SLNB: Sentinel lymph node biopsy
TN: Triple negative
TNBC: Triple negative breast cancer
AI: Aromatase inhibitor
SP: Seguro popular
ET: Endocrine therapy
HR: Hormone receptor
ER: Estrogen receptor
PR: progesterone receptor.
