# Supplementary material for: Access to care issues adversely affect breast cancer patients in Mexico: oncologists’ perspective
Source: BMC Cancer. 2014 Sep 9;14:658. doi: 10.1186/1471-2407-14-658 (PMC4165903; doi:10.1186/1471-2407-14-658)
Supplement: Supplementary file 1 — Additional file 1: Figure S1: Oncology physician patterns of practice survey. (DOCX 23 KB) [file 12885_2013_4840_MOESM1_ESM.docx]

**Additional file 1: Figure S1**

**Breast Physician Practice Survey**

Please complete the survey below.

Thank you!

Please complete this survey if you are a practicing physician who treats breast cancer patients with hormonal therapy or chemotherapy. You do not need to complete the survey at one time. You may save your progress and return at a later time. After you have finished, click SUBMIT.

**Personal Information**

1. How many patients with newly diagnosed breast cancer do you care for on average each month? (Choose one answer)

- <5
- 5-10
- 11-20
- 21-30
- 31-40
- 40-50
- >50

2. Please specify your gender (Choose one answer)

- Female
- Male

3. Please specify your age (Choose one answer)

- < 40
- 40-65
- 65

4. Please indicate the country in which you primarily practice medicine: (Choose one answer)

- Argentina
- Australia
- Belgium
- Bhutan
- Brazil
- Canada
- China
- Chile
- Croatia
- Czechoslovakia
- Denmark
- France
- Georgia
- Germany
- Greece
- Guatemala
- Hong Kong
- Hungary
- India
- Israel
- Italy
- Latvia
- Lithuania
- Mexico
- New Zealand
- Peru
- Philippines
- Poland
- Russia
- Slovakia
- South Africa
- South Korea
- Spain
- Thailand
- Turkey
- Ukraine
- United Kingdom
- United States
- Other

5. In what State/Region/Province is your Medical Practice?

_____________________________

6. Please tell us the number of years since you received your medical degree: (Choose one answer)

- 1-5
- 6-10
- 11-20
- 21-30
- Over 30

7. In what sub-specialty did you primarily train (or are in training)? (Choose one answer)

- Medical Oncology
- Hematology
- Radiation Oncology
- Surgical Oncology
- Breast Surgeon
- Internal Medicine
- Other (please specify)

If other please specify:________________

8. In what type of geographic area do you practice (Choose one answer)

- Urban center
- Suburban area
- Rural area
- Other (please specify)

If other, please specify: ________________________

9. Where is your primary practice located? (Choose one answer)

- Academic medical center/University
- Public Hospital/Clinic
- Philanthropic hospital or clinic
- Private hospital/clinic
- Other

If other, please specify __________________________________

10. Of all the breast cancer patients you attend, please give the percent who use each of the following payment methods.

Private out-of-pocket patients: ____%

Private health insurance patients _____%

Public health insurance patients _____ %

Patients without any insurance or out-of-pocket means ____%

Other _____ %

If other, please specify __________________________________

11. Based on the patients you commonly see in your practice, please estimate (in percentages) the INITIAL CLINICAL BREAST CANCER STAGE of your NEW patients at disease presentation:

Stage I- II breast cancer _________%

Stage III breast cancer _________%

Stage IV breast cancer _________%

**Based on the most common type of patients in your practice, please tell us about PATHOLOGY REPORT INFORMATION:**

12. In a regular pathology report that you receive, please check all that are usually available to you? (Check all that apply)

- Tumor Size
- Tumor Grade
- Presence/absence of vascular invasion
- Margin Status
- Lymph Node Analysis
- Estrogen receptor
- Progesterone receptor
- HER2/neu

13. To what method of HER2/neu testing do your patients routinely have access? (Check all that apply)

- Immunhohistochemistry (IHC)
- Fluorescence in situ hybridization (FISH)
- Other (please specify)
- HER2/neu testing is not routinely done for my patients

If other selected, please specify: ­­­­­­­­­­­­__________________________

14. For the patients that get HER2/neu testing, where is it done? (Choose one answer)

- At my own hospital/center
- At an outside facility at a central location
- Other (specify)

If other, please specify: **­­­­­­­­­­­**______________________________

**Based on the most common type of patients in your practice, please tell us about**

**THERAPY FOR LOCALIZED BREAST CANCER:**

15. Please estimate the percentage of newly diagnosed patients that undergo mastectomy ___%

15b.Please estimate the percentage of newly diagnosed patients that undergo lumpectomy ___%

16. Please estimate the percentage of newly diagnosed patients without palpable axillary lymph nodes who undergo sentinel lymph node sampling ___%

16b. Please estimate the percentage of newly diagnosed patients without palpable axillary lymph nodes who undergo axillary lymph node sampling without prior sentinel lymph nodes sampling ___%

17. Do you have ADJUVANT RADIOTHERAPY available to your patients? (Choose one answer)

- Yes
- No

18. If yes, what kind of radiotherapy do your patients routinely receive? (Choose one answer)

- Once Daily Fractionated Radiotherapy in 5-6 weeks
- Intraoperative Radiotherapy
- Accelerated Partial Breast Radiotherapy
- Other (please describe)

If other, please describe: ­­­­­______________________

19. In what percentage of your patients do you use neoadjuvant therapy?

Stage I___%

Stage II ___%

Stage III ____%

20. What is the average time interval between definitive surgery and start of adjuvant chemotherapy for your patients?

- <3 weeks
- 3-12 weeks
- >12 weeks

**The following questions ask about how access to care impacts breast cancer treatment. Please check at least 1 box in each question.**

**Questions 21-24 refer to THERAPY FOR ESTROGEN RECEPTOR POSITIVE breast cancer.**

**For the types of patients below, indicate recommended treatment with free access to any medication vs. actual treatment based on your current access to treatment.**

21. Patient with LOW RISK (HER2/neu NEGATIVE, < 1cm in size and NODE NEGATIVE): Treatment Recommendation Given FREE ACCESS to any Medication (choose all that apply)

- Endocrine therapy
- Anthracycline-Non taxane
- Anthracycline-taxane
- Taxane- Non-Anthracycline
- Other (please specify)

If other, please specify: __________________________

21b. Patient with LOW RISK (HER2/neu NEGATIVE, < 1cm in size and NODE NEGATIVE): Treatment Recommendation Given CURRENT ACCESS to Care (choose all that apply)

- Endocrine therapy
- Anthracycline-Non taxane
- Anthracycline-taxane
- Taxane- Non-Anthracycline
- Other (please specify)

If other, please specify: __________________________

22. Patient with HIGH RISK (HER2/neu NEGATIVE, > 1cm in size and NODE POSITIVE): Treatment Recommendation Given FREE ACCESS to any Medication (choose all that apply)

- Endocrine therapy
- Anthracycline-Non taxane
- Anthracycline-taxane
- Taxane- Non-Anthracycline
- Other (please specify)

If other, please specify: __________________________

22b. Patient with HIGH RISK (HER2/neu NEGATIVE, > 1cm in size and NODE POSITIVE): Treatment Recommendation Given CURRENT ACCESS to Care (choose all that apply)

- Endocrine therapy
- Anthracycline-Non taxane
- Anthracycline-taxane
- Taxane- Non-Anthracycline
- Other (please specify)

If other, please specify: __________________________

23. Patient that is PRE-menopausal: Treatment Recommendation Given FREE ACCESS to any Medication (choose all that apply)

- Ovarian suppression
- Tamoxifen: specify length in years _____
- Aromatase inhibitor: specify length in years _____
- Other (please describe)

If you marked 'other' above, please specify: _______________________________________

Please specify the length of Tamoxifen (in years): __________________________________

Please specify the length of Aromatase Inhibitor (in years) __________________________

23b. Patient that is PRE-menopausal: Treatment Recommendation Given CURRENT ACCESS to Care (choose all that apply)

- Ovarian suppression
- Tamoxifen: specify length in years _____
- Aromatase inhibitor: specify length in years _____
- Other (please describe)

If you marked 'other' above, please specify: _______________________________________

Please specify the length of Tamoxifen (in years): __________________________________

Please specify the length of Aromatase Inhibitor (in years) __________________________

24. Patient that is POST-menopausal: Treatment Recommendation Given FREE ACCESS to any Medication (choose all that apply)

- Ovarian suppression
- Tamoxifen: specify length in years _____
- Aromatase inhibitor: specify length in years _____
- Other (please describe)

If you marked 'other' above, please specify: _______________________________________

Please specify the length of Tamoxifen (in years): __________________________________

Please specify the length of Aromatase Inhibitor (in years) __________________________

24b. Patient that is POST-menopausal: Treatment Recommendation Given CURRENT ACCESS to Care (choose all that apply)

- Ovarian suppression
- Tamoxifen: specify length in years _____
- Aromatase inhibitor: specify length in years _____
- Other (please describe)

If you marked 'other' above, please specify: _______________________________________

Please specify the length of Tamoxifen (in years): __________________________________

Please specify the length of Aromatase Inhibitor (in years) __________________________

**Questions 25-26 refer to THERAPY FOR TRIPLE NEGATIVE breast cancer:**

**For the types of patients below, indicate recommended treatment with free access to any medication vs. actual treatment based on your current access to treatment.**

25. Patient that has a TUMOR < 1 cm and NEGATIVE NODES: Treatment Recommendation Given FREE ACCESS to any Medication (choose one answer)

- No Adjuvant treatment
- Anthracycline-Non taxane combination
- Anthracycline-taxane combination
- Taxane-Non Antracycline combination
- Other (please specify)

If other, please specify: ____________________________

25b. Patient that has a TUMOR < 1 cm and NEGATIVE NODES: Treatment Recommendation Given CURRENT ACCESS to care. (choose one answer)

- No Adjuvant treatment
- Anthracycline-Non taxane combination
- Anthracycline-taxane combination
- Taxane-Non Antracycline combination
- Other (please specify)

If other, please specify: ____________________________

26. Patient that has a TUMOR > 1 cm and POSITIVE NODES: Treatment Recommendation Given FREE ACCESS to any Medication (choose one answer)

- No Adjuvant treatment
- Anthracycline-Non taxane combination
- Anthracycline-taxane combination
- Taxane-Non Antracycline combination
- Other (please specify)

If other, please specify: ____________________________

26b. Patient that has a TUMOR > 1 cm and POSITIVE NODES: Treatment Recommendation Given CURRENT ACCESS to care. (choose one answer)

- No Adjuvant treatment
- Anthracycline-Non taxane combination
- Anthracycline-taxane combination
- Taxane-Non Antracycline combination
- Other (please specify)

If other, please specify: ____________________________

**Questions 27-28 refer to THERAPY FOR HER2 POSITIVE breast cancer:**

**For the types of patients below, indicate recommended treatment with free access to any medication vs. actual treatment based on your current access to treatment.**

27. Patient with a ER POSITIVE, < 1 cm and NODE NEGATIVE tumor: Treatment Recommendation Given Free Access to any Medication (check all that apply)

- Endocrine treatment
- Trastuzumab
- Chemotherapy
- Other (please specify)

If other, please specify: ____________________________

27b. Patient with a ER POSITIVE, < 1 cm and NODE NEGATIVE tumor: Treatment Recommendation Given Current Access to Care (check all that apply)

- Endocrine treatment
- Trastuzumab
- Chemotherapy
- Other (please specify)

If other, please specify: ____________________________

28. Patient with a ER NEGATIVE, > 1 cm and NODE POSITIVE tumor: Treatment Recommendation Given Free Access to any Medication (check all that apply)

- Endocrine treatment
- Trastuzumab
- Chemotherapy
- Other (please specify)

If other, please specify: ____________________________

28b. Patient with a ER NEGATIVE, > 1 cm and NODE POSITIVE tumor: Treatment Recommendation Given Current Access to Care (check all that apply)

- Endocrine treatment
- Trastuzumab
- Chemotherapy
- Other (please specify)

If other, please specify: ____________________________

29. Within the last year, have there been instances where you would like to have recommended adjuvant trastuzumab but the patient did not ultimately receive it? (Choose one answer)

- Yes
- No

30. If you have answered YES, please mark the reasons why the patient did not receive trastuzumab: (Check all that apply)

- Patient Refusal
- Public health care without coverage
- Private health care without coverage
- Cost for out-of-pocket payment too high
- Preferred a clinical trial
- Another practitioner felt herceptin was not indicated
- Unable to make the trip and visits necessary for treatment
- Patient co-morbidities that raised concern about added toxicities
- Other (Please Specify)

If other, please specify: ____________________________

**Please tell us about availability of CLINICAL TRIALS where you work:**

31. Are there ongoing clinical trials for breast cancer treatment that are actively enrolling patients at or near your primary practice? (Choose one answer)

- Yes
- No (skip next question)
- I don’t know (skip next question)

32. Do you regularly send patients for enrollment in these clinical trials? (Choose one answer)

- Yes
- No

33. Do you feel that you are currently able to give your patients the best treatment available? (Choose one answer)

- Yes (skip next question)
- No

34. If NO, why? (Check all that apply)

- Delay in pathology reports
- Pathology reports without important prognostic/predictive information
- Restrictions for prescribing ideal chemotherapy protocols
- Delay in patient beginning chemotherapy after prescription
- Restrictions in prescribing ideal hormonal therapy
- Restrictions in prescribing Trastuzumab therapy for HER2+ tumors
- My workload is too high
- Other (please describe)

If other, please specify __________________________________

THANK YOU FOR PARTICIPATING!
